# Development of anti-cancer drugs for tumor-associated macrophages: a comprehensive review and mechanistic insights

**DOI:** 10.3389/fmolb.2024.1463061

**Published:** 2024-12-09

**Authors:** Bingjun Bai, Shangzhi Xie, Ya Wang, Fei Wu, Yao Chen, Jia Bian, Xing Gao

**Affiliations:** ^1^ Department of Colorectal Surgery, Sir Run Run Shaw Hospital, School of Medicine, Zhejiang University, Hangzhou, China; ^2^ Institute of Genomic Medicine, Wenzhou Medical University, Wenzhou, China; ^3^ Department of Hospital Infection-Control, Zhejiang Cancer Hospital, Hangzhou, China; ^4^ Institute of Systemic Medicine, Zhejiang University School of Medicine, Hangzhou, China; ^5^ Department of Medical Oncology, Sir Run Run Shaw Hospital, School of Medicine, Zhejiang University, Hangzhou, China; ^6^ Department of Gynecology and Obstetrics, The Affiliated People’s Hospital of Ningbo University, Ningbo, Zhejiang, China; ^7^ Department of Oncology, The Second Affiliated Hospital of Soochow University, Suzhou, Jiangsu, China

**Keywords:** tumor-associated macrophages (TAMs), tumor microenvironment (TME), TAMs-targeted therapies, macrophage polarization, M1 and M2 macrophages

## Abstract

This review provides an in-depth summary of the development of anti-cancer drugs for tumor-associated macrophages (TAMs), with a particular focus on the development and tissue specialization of macrophages, and factors influencing the polarization of M1 and M2 macrophages, and mechanistic insights underlying the targeting therapeutic approaches. TAMs, pivotal in the tumor microenvironment, exhibit notable plasticity and diverse functional roles. Influenced by the complex milieu, TAMs polarize into M1-type, which suppresses tumors, and M2-type, which promotes metastasis. Notably, targeting M2-TAMs is a promising strategy for tumor therapy. By emphasizing the importance of macrophages as a therapeutic target of anti-cancer drugs, this review aims to provide valuable insights and research directions for clinicians and researchers.

## 1 Introduction

### 1.1 Macrophages: development and tissue specialization

Macrophages, initially detailed by Ilya Metchnikoff (Stefater et al., 2011), possess the capacity to engulf and eliminate cellular components from both living and dead organisms and host cells (Ji et al., 2024). Macrophages can be categorized into two main subtypes: tissue-resident macrophages and monocyte-derived inflammatory macrophages. Tissue-resident macrophages primarily execute anti-tumor functions through cytotoxicity and antibody-dependent cell-mediated cytotoxicity (ADCC). In contrast, monocyte-derived inflammatory macrophages tend to promote tumor metastasis and suppress T cell-mediated immune responses (Sun et al., 2023). The predominant population, tissue-resident macrophages, originates embryonically and is distributed across various tissues where microcellular invasion or foreign body accumulation occurs frequently, such as the liver, lymph nodes, and spleen. Recent research has highlighted the indispensable role of tissue-resident macrophages in responding to lung injury post-trauma or stroke through specific inflammatory pathways (Aegerter et al., 2022). Notably, investigations have revealed that depleting tissue-resident macrophages, instead of impeding the recruitment of monocyte-derived macrophages, effectively mitigates lung injury after trauma or stroke events (Hoyer et al., 2019). Over time, the belief that tumor-associated macrophages (TAMs) were primarily recruited from peripheral sites has shifted due to novel insights from gene pedigree tracing, xenogeneic reproduction, and bone marrow chimera studies, indicating both embryonic and pathological origins as key sources (1) (Rodell et al., 2019; Fu et al., 2020). In a steady state, most tissue-resident macrophages are derived from the yolk sac and fetal liver. However, during pathological conditions, monocytes turned out to be a prominent source (van de Laar et al., 2016). These findings about mature macrophage proliferation prompt us to re-evaluate the interplay between macrophage proliferation and differentiation. Each organ harbors a unique macrophage subpopulation that, following birth, is renewed by circulating monocytes, establishing a distinct regeneration pattern.

### 1.2 Tumor microenvironment and tumor-associated macrophages

The tumor microenvironment (TME), consisting of various cellular and non-cellular components, is the complex ecosystem that surrounds and interacts with cancer cells within a tumor. As one of the most concerned cellular components, immune cells include innate immune system cells (including macrophages, neutrophils, myelogenic suppressor cells, dendritic cells and natural killer cells) and adaptive immune system cells (T and B lymphocytes) (Ji et al., 2024; Anderson and Simon, 2020; Ruf et al., 2023). The TME is highly dynamic and can influence cancer cell behavior, drug resistance, and treatment outcomes (Escamilla et al., 2015). Understanding and targeting the TME has become an important focus in cancer research and therapy development, leading to new approaches such as immunotherapy and strategies to modify the tumor microenvironment to enhance treatment efficacy (Ruf et al., 2023).

Tumor metastasis, a leading cause of cancer-related deaths, is propelled not only by intrinsic alterations in tumor cells but also by the intricate interactions between cancer cells and their evolving microenvironment (Neophytou et al., 2021). TAMs are crucial in these interactions, orchestrating the production of cytokines, chemokines, and growth factors by T cells, while also promoting the release of inhibitory immune checkpoint proteins that foster an immunosuppressive microenvironment (Sun et al., 2023). Although evidence indicates that various types of macrophages can coexist in tumors, recruited macrophages may account for the majority of TAMs (Bied et al., 2023). By influencing cancer cell metastasis and offering targets for immunotherapy, TAMs play a central role in tumor progression. Simultaneously, they offer several targets for blocking immunotherapy at some checkpoints to combat tumor progression. As their relationship with malignant tumors becomes clearer, TAMs are garnering recognition as potential biomarkers for cancer diagnosis and prognostic assessment. Moreover, they have emerged as promising therapeutic targets in cancer treatment, spurring active research in this field (Dallavalasa et al., 2021). However, quantifying the respective contributions of these macrophage subtypes at different stages of tumor progression remains challenging. Therefore, further investigations aimed at characterizing TAMs across different human cancers are warranted (Dallavalasa et al., 2021).

### 1.3 Factors influencing the polarization of macrophages

Macrophage polarization is a complex biological process intricately governed by a multitude of factors, with cytokines and signaling pathways playing pivotal roles. Pathways such as PI3K/Akt, MAPK, and NF- κB are central to this regulatory network (Hu et al., 2002; Jia et al., 2023; Tosic and Frank, 2021). Macrophages express various markers from different subtypes depending on their microenvironment and activation state so that their classifications are not rigid. CD68 is a pan-macrophage marker widely used to identify macrophages in human tissues but lacks specificity. CD80 and CD86 are co-stimulatory molecules expressed on activated macrophages, which are often associated with pro-inflammatory (M1) macrophages (Van Dyken and Locksley, 2013). In contrast, CD206 is another marker associated with anti-inflammatory (M2) macrophages. Besides, CD163, a number of the scavenger receptor family, offers greater specificity and is exclusive to the monocyte/macrophage lineage (Tardito et al., 2019). CD115, also named the receptor for colony-stimulating factor 1 (CSF1R), is important for macrophage development. CD204 is expressed on tissue macrophages. What’s more, advanced techniques like flow cytometry, immunohistochemistry, and single-cell RNA sequencing are used to analyze these markers and characterize macrophage populations in complex tissues or cell mixtures.

**FIGURE 1:**
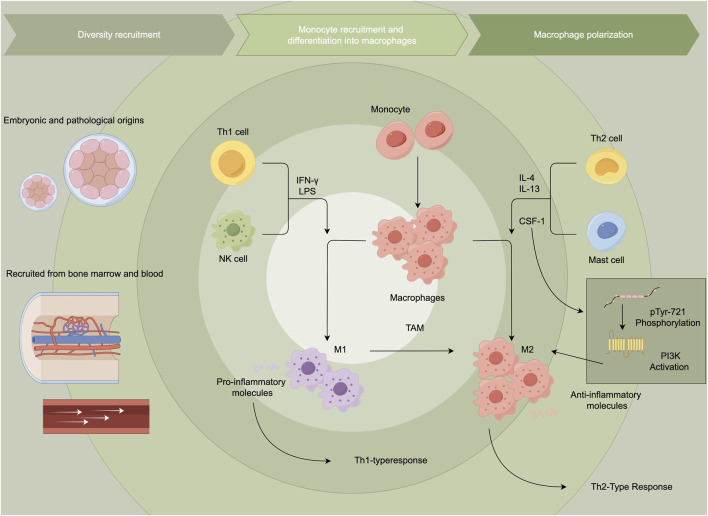
Tumor-associated macrophages and macrophage polarization. This figure illustrates the recruitment and differentiation of monocytes into macrophages, highlighting their polarization into pro-inflammatory M1 or anti-inflammatory M2 phenotypes. M1 macrophages promote Th1 responses, while M2 macrophages, often associated with tumor-associated macrophages, support Th2 responses and tumor progression.

Researchers often use combinations of several markers to more accurately characterize macrophage populations in specific circumstances. The M1/M2 classification is a simplification of macrophage activation states. These two distinct types of macrophages have different functions and characteristics, and they play crucial roles in various physiological and pathological processes. M1 macrophages, also known as “Killer” macrophages, differentiate from monocytes in response to type 1 T helper cell (Th1) cytokines like INF-γ, granulocyte-macrophage colony-stimulating factor and lipopolysaccharide (Pasca et al., 2020). M1 macrophages are involved in the pro-inflammatory response, host defense against pathogens, and anti-tumor activities. They produce high levels of pro-inflammatory cytokines, such as interleukin (IL)-1, IL-6, IL-12, IL-23, and tumor necrosis factor (TNF) (Klichinsky et al., 2020). Besides, M1 macrophages produce high levels of nitric oxide through expressing inducible Nitric Oxide Synthase (iNOS). In contrast, M2 macrophages, termed “Repair” macrophages, are activated by anti-inflammatory cytokines, such as IL-4, IL-13, IL-10, macrophage colony-stimulating factor (M-CSF), prostaglandin F (PGF), and vitamin D3. The main functions of M2 macrophages are tissue repair, wound healing, and immunoregulation. They mainly produce anti-inflammatory cytokines such as IL-10, TGF-β, VEGF-A, and matrix metalloproteinase 2 (MMP2). A higher expression level of arginase-1 enables M2 macrophages to have the function of tissue repair and fibrosis. M2 macrophages promote angiogenesis by producing growth factors, like VEGF. At the level of transcriptional modification, most changes in histone methylation and acetylation modifiers are concentrated in M1 macrophages (Zhang et al., 2024), for example, histone acetylation mediated by acetyl-CoA enhances the expression of pro-inflammatory genes in LPS-activated macrophages (Sun et al., 2022), while almost all modifiers related to histone crotonylation are activated in M2 macrophages. In the tumor microenvironment, TAMs are commonly categorized as M2-like macrophages, distinguished by elevated levels of anti-inflammatory cytokines, scavenger receptors, angiogenic factors, and proteases. The signaling axis of CSF-1 and its receptor CSF-1R remarkably influence the survival and activation of TAMs. Tumor cells secrete CSF-1, which not only promotes the recruitment of macrophages but also polarizes them towards an M2-like TAM phenotype (Pedersen et al., 2014). When CSF-1R is activated, phosphorylation at the Y721 site (pTyr-721) provides a critical binding and activation site for PI3K (Figure 1) (Guo et al., 2006; Caescu et al., 2015).

Across various cancer types, including non-small cell lung cancer (NSCLC), ovarian, gastric, melanoma, and breast cancers, CD68 expression is associated with a poorer prognosis and reduced survival rates (Zhang et al., 2017). Specifically in NSCLC, CD68-positive macrophages inversely correlate with patient survival and are associated with increased tumor IL-8 expression. This relationship suggests a potential contribution to heightened tumor angiogenesis (Zhang et al., 2017). Elevated levels of CD68-positive infiltrating TAMs in gastric cancer (GC) are associated with increased metastasis and poor prognostic outcomes. Moreover, these TAMs also exhibit features of epithelial-mesenchymal transition (EMT), characterized by the loss of E-cadherin expression and positivity for vimentin (Yang et al., 2019). Similarly, in melanoma, an abundance of CD68+ macrophages correlates with a poorer prognosis and increased melanoma-specific mortality (Tariq et al., 2017). Zhang et al. suggest that the reduction of CD206+ TAMs in gastric cancer is associated with prolonged disease-free survival (DFS), highlighting their significance as prognostic indicators (Zhang et al., 2021). Recent studies also reveal a notable positive correlation between the infiltration of CD206-expressing TAMs in ovarian and renal cancers and lower patient survival rates (Sun et al., 2020). Stabilin-1, a multifunctional scavenger receptor found in alternatively activated macrophages, is critical for removing unwanted autologous material from the body (Manta et al., 2022). Extensive infiltration of Stabilin-1-expressing TAMs has been observed in metastatic lesions of human primary breast tumors. Notably, these TAMs show tumor growth-promoting properties in a mouse model of mammary adenocarcinoma (Riabov et al., 2016).

## 2 Therapeutic target of cancer

TAMs are instrumental in enhancing tumor cell resistance to chemotherapy and radiotherapy by delivering survival factors and activating anti-apoptotic mechanisms. Consequently, anticancer therapeutic targeting TAMs have a solid theoretical basis and are expected to be a promising strategy for enhancing therapeutic efficacy. Approaches to target TAMs include: 1) inhibiting monocyte recruitment from systemic circulation to tumor sites; 2) reprogramming TAMs towards an anti-tumor phenotype; 3) disrupting TAM activation pathways to reduce their pro-tumor activities; and 4) integrating TAM targeting with conventional treatments like chemotherapy or radiotherapy (Anfray et al., 2019).

### 2.1 Inhibition of monocyte induction from systemic circulation to the tumor tissue

Tumor cells promote the migration of myeloid-derived suppressor cells (MDSCs) and macrophages into the tumor microenvironment by secreting chemokines such as CCL2, CCL5, and CXCL12. Disrupting these recruitment pathways can effectively slow tumor growth and progression (Groth et al., 2019). Carlumab, a monoclonal antibody targeting CCL2, has shown efficacy in reducing prostate-specific antigen levels and inhibiting tumor progression in a phase II clinical trial (Martori et al., 2022). Similarly, Bindarit inhibits key inflammatory chemokines including MCP-1 (CCL2), MCP-3 (CCL7), and MCP-2 (CCL8), significantly affecting the NF-κB signaling pathway associated with monocyte recruitment without impacting other pathways, demonstrating its specificity and potential in reducing monocyte recruitment into tumors (Wolfsberger et al., 2021).

Inhibiting monocyte induction from systemic circulation to tumor tissue is a pivotal strategy in cancer treatment. Targeting the chemokine signaling pathways that facilitate monocyte recruitment offers a promising approach to alter the tumor microenvironment and potentially boost the efficacy of cancer therapies. Table 1 below highlights several drugs currently explored for their role in inhibiting monocyte induction and influencing TAM behavior across various cancer types: BLZ945 and PLX3397, targeting CSF-1R, are crucial in modulating the tumor microenvironment by reducing monocyte recruitment and enhancing T cell infiltration. These interventions, evidenced in trials NCT02829723 and NCT02371369, not only impede tumor progression but also strengthen the overall immune response within the tumor milieu. Cabiralizumab, another CSF-1R inhibitor, disrupts monocyte recruitment pathways, significantly impacting the development and progression of solid tumors as indicated in clinical trial NCT03502330. CCX872 targets CCR2, playing a significant role in monocyte recruitment to tumor sites. By blocking this pathway, CCX872 aims to reduce the number of macrophages in the tumor microenvironment, potentially limiting tumor growth and metastasis, particularly in pancreatic cancer, as studied in NCT02345408.

**TABLE 1:** Proposed tumor-associated macrophages targeting therapy.

Drug/Compound	Target	Tumor type	Phase	Clinical trials
BLZ945	CSF-1R	Advanced solid tumors	I/II	NCT02829723
PLX3397	CSF-1R	Tenosynovial giant cell tumor	III	NCT02371369
Cabiralizumab	CSF-1R	Advanced solid tumors	I	NCT03502330
Biophosphonates	TAMs	Various solid tumors	II	Multiple trials
Ibrutinib	BTK	Various cancers	II/III	Multiple trials
CCX872	CCR2	Pancreatic cancer	I/II	NCT02345408
Trabectedin	TAMs	Soft tissue sarcoma	III	Various trials exploring

### 2.2 Reprogramming of TAMs

Reprogramming TAMs to adopt an anti-tumor phenotype represents a promising avenue in cancer immunotherapy. This approach aims to shift their behavior from pro-tumor (M2-like) to anti-tumor (M1-like) activities, thereby harnessing the immune system’s natural defenses against cancer. Various therapeutic strategies have been developed to achieve this goal, including the use of CSF1R inhibitors, immune checkpoint inhibitors, and other reprogramming agents. This intervention works by encouraging TAMs to enhance immune cell recruitment, augment phagocytic activity, and produce cytotoxic molecules that directly attack tumor cells. By remodeling the tumor microenvironment towards an immune-supportive state, these approaches not only suppress tumor growth but also synergistically enhance the efficacy of existing cancer treatments, potentially leading to improved clinical outcomes for patients (Genard et al., 2017; Khan et al., 2023).

Thymosin-α, a small bioactive polypeptide secreted by thymus tissue, has garnered significant attention in cancer immunotherapy due to its unique capacity to reprogram TAMs into functional dendritic cells. This transformation profoundly impacts T cell differentiation and activation, ultimately amplifying the anti-tumor immune response (Sun et al., 2023). This reprogramming is leveraged in treatments for metastatic melanoma and advanced non-small cell lung cancer (NSCLC). Clinical studies have demonstrated that Thymosin-α-based therapies can significantly prolong patient survival, offering a promising adjunct to conventional cancer treatments. Moreover, its capacity to modulate the tumor microenvironment and enhance immune surveillance has sparked interest in combining Thymosin-α with other immunotherapeutic approaches, potentially opening new avenues for more effective and personalized cancer management strategies (Singh et al., 2017). Overall, the reprogramming of TAMs represents a promising area in cancer therapy, focusing on converting the immunosuppressive tumor environment into an immunostimulatory one.

### 2.3 Targeting the activation of TAMs

Legumain, a lysosomal cysteine protease, is highly expressed in many solid tumors, TAMs and endothelial cells of tumor neovascularization. Angiogenesis, tumor invasion, proliferation, and metastasis are pivotal events in malignant tumor progression, intricately linked to various biological processes within the tumor microenvironment. Recent studies have unveiled that a DNA vaccine targeting legumain can effectively stimulate CD8^+^ T cells to attack TAMs, leading to a significant reduction in TAM density within tumor tissues. Furthermore, this vaccine markedly decreases the release of multiple angiogenic factors from TAMs, including transforming growth factor-β (TGF-β), tumor necrosis factor-α (TNF-α), matrix metalloproteinase-9 (MMP-9), and vascular endothelial growth factor (VEGF). This approach holds the potential to inhibit tumor angiogenesis and metastasis, presenting a novel strategy for cancer treatment. Therefore, Legumain based DNA vaccine can effectively inhibit the growth and metastasis of tumor cells in mouse models of breast cancer, non-small cell lung cancer and colon cancer (Ngambenjawong et al., 2017). Furthermore, CD200S, a variant of CD200, plays a significant role in the reprogramming of TAMs by inducing their trans-differentiation into dendritic cells. This alteration enhances immune responses against tumor cells, effectively targeting the activation pathways of TAMs to curb tumor growth, as shown in a mouse glioma model (Liu et al., 2020).

Recent research highlights the novel application of lovastatin in cancer therapy, particularly its capacity to reduce the population of TAMs. Through gene chip analysis, lovastatin has been observed to downregulate the expression of placental growth factor (PlGF), which is closely associated with enhanced TAM activity. Thus, beyond its conventional role in managing cholesterol, lovastatin presents a new avenue for modulating anti-tumor immunity, offering a fresh strategy for cancer treatment (Yang et al., 2020). Moreover, the role of protein deacetylases in regulating TAM activity has been emphasized in studies involving TMP195, a small molecule inhibitor of class IIa histone deacetylase. Research using the MMTV-PyMT transgenic breast cancer model demonstrated that TMP195 not only stabilizes tumor volume but also significantly reduces the incidence of lung metastases by about threefold. This suggests that by altering TAM functions, TMP195 induces profound changes in the tumor microenvironment, effectively inhibiting tumor growth and the spread of metastasis. These outcomes support the potential of targeted immunotherapy in cancer treatment (Lopez-Yrigoyen et al., 2021).

Nuclear Factor-kappa B (NF-κB) plays a critical role in regulating inflammatory responses, which are heavily implicated in various tumor processes such as promoting Epithelial-Mesenchymal Transition (EMT), enhancing tumor cell migration, and generating Tumor-Initiating Stem Cells (TSCs) (Zhu et al., 2021). These activities collectively contribute to the malignant progression and metastatic potential of tumors. Importantly, TAMs, influenced by NF-κB signals, are known to promote and mediate angiogenesis through the production of interleukins like IL-10, VEGF, and IL-8, making NF-κB a viable target for treating cancers such as urinary bladder cancer (UBC) (Iacona and Lutz, 2019). BAY11-7082, an inhibitor of the NF-κB pathway, effectively reduces the invasiveness of bladder cancer cells and prevents the M2 polarization of TAMs, highlighting its role in macrophage polarization and its potential in modulating tumor microenvironments (Zhang et al., 2019).

### 2.4 Targeting TAMs in combination with standard therapies

Signal regulatory protein α (SIRP α) is a membrane protein primarily expressed on macrophages and other myeloid immune cells. It can be activated by various mitogens and phosphorylated, transmitting inhibitory signals through binding to SHP-1 and SHP-2 via its immunoreceptor tyrosine-based inhibition motif (ITIM) domain. Additionally, SIRP α can inhibit the activation of downstream pathways and convey negative signals by interacting with CD47 ligands. The blockers of exosomes SIRP α and CD47 can enhance the phagocytic function of cancer cells, suggesting that targeting SIRP α with antibodies presents a promising immunotherapy approach for treating tumors exhibiting high expression of SIRP α, such as renal cell carcinoma and melanoma (Yanagita et al., 2017). Anti-CD47 antibodies are currently demonstrating significant therapeutic potential as agents targeting the CD47/SIRP α signaling axis. Several CD47 antagonists are undergoing extensive investigation in clinical trials, including Hu5F9-G4, humanized anti-human CD47 monoclonal antibody CC-90002, and TTI-621. These drugs operate through various mechanisms to obstruct the interaction between CD47 and SIRP α, thereby stimulating the phagocytosis of tumor cells by macrophages. Consequently, they enhance the immune system’s ability to combat tumors, offering novel strategies for treating multiple types of cancer (Weiskopf, 2017; Folkes et al., 2018). Humanized anti-human CD47 monoclonal antibody CC-90002 is currently under investigation for use in both solid tumors and hematological malignancies. TTI-621 is composed of the Ig-V-like domain of human SIRP α connected to the Fc region of human IgG1, which can enhance the phagocytosis of tumor cells and effectively control tumor growth. The potential of targeting CD47 lies in its combination with immunotherapy agents like PD-1 antibodies, aiming to maximize its therapeutic efficacy (Ansell et al., 2021; Feng et al., 2019).

Metformin, a biguanide widely used in diabetes management, has gained attention for its potential applications in the field of antitumor therapy. This effect is primarily attributed to the activation of the AMP-activated protein kinase (AMPK) signaling pathway, which disrupts the M2 polarization of macrophages. These findings suggest that metformin may exert an anti-tumor metastatic effect, providing a new scientific rationale for its use in anti-tumor therapy. Moreover, these discoveries may further expand the therapeutic potential of metformin in tumor populations (Neophytou et al., 2021).

Resveratrol, a non-flavonoid polyphenolic compound, has garnered significant attention due to its diverse biological activities, including its role as an antioxidant, anti-inflammatory, and anti-cancer agent. It has been observed to inhibit the activation of M2-type macrophages, impacting the tumor microenvironment significantly. One of its mechanisms involves the reduction of STAT3 activation and the decrease in F4/80-positive cells, which contributes to its anti-tumor effects, particularly in inhibiting lung cancer growth. However, the complexities of resveratrol’s mechanisms require further investigation to fully understand and harness its potential in cancer therapy (Ge and Ding, 2020).

A novel and promising therapeutic approach for targeting TAMs within the tumor microenvironment involves the use of Chimeric Antigen Receptor T (CAR-T) cells. While traditional CAR-T therapies have been effective in hematological malignancies, the application of this technology to solid tumors has faced significant challenges. However, recent advancements have focused on engineering CAR-T cells to specifically target TAMs, which play a key role in promoting tumor growth and suppressing anti-tumor immunity. By utilizing CAR-T cells directed against macrophage markers such as F4/80, researchers have demonstrated significant reductions in TAM populations within tumors, leading to delayed tumor progression and enhanced immune responses. Importantly, these CAR-T cells not only deplete TAMs but also trigger the release of cytokines, such as IFN-γ, which reprogram the tumor microenvironment to favor immune cell infiltration and activation. This innovative strategy holds promise for improving outcomes in solid tumors by targeting the immunosuppressive components of the tumor microenvironment, potentially paving the way for more effective immunotherapies in the future (Sánchez-Paulete et al., 2022).

Recent advances have introduced CAR-T cell therapies targeting specific TAM markers, such as F4/80, as a novel treatment strategy. This approach not only depletes TAM populations but also reprograms them toward a pro-inflammatory (M1-like) state, enhancing anti-tumor immune responses (Xiang et al., 2021).

Current trends in cancer treatment research increasingly emphasize the development of integrative therapeutic approaches that combine chemotherapy with immunotherapy. For instance, the combination of immune checkpoint inhibitors targeting PD-1, PD-L1, and CTLA-4 with traditional chemotherapies has shown significant survival benefits in treating metastatic tumors, marking substantial progress in oncological therapeutics (Larionova et al., 2019).

Paclitaxel, a naturally derived anticancer drug, has gained wide application in treating various cancers, including breast, ovarian, and lung cancer. By combining paclitaxel with a CSF1R signaling antagonist, studies have shown an effective reduction in macrophage recruitment to tumor sites, thereby enhancing the drug’s effectiveness and reducing tumor progression and metastasis in preclinical models (Mantovani et al., 2022). Moreover, this combination has been demonstrated to improve the efficacy of paclitaxel on breast tumors and extend survival in a mouse model with mammary tumors, highlighting the potential of TAM-targeted combination therapies in clinical settings (Brown et al., 2017).

Recent advancements have shown that targeting tumor-associated macrophages can significantly enhance the effectiveness of gemcitabine, particularly in treating pancreatic cancer and NSCLC (Rohila et al., 2023). By inhibiting CSF1R or targeting the CCR2 receptor, both key to macrophage recruitment in tumors, the therapeutic effectiveness of gemcitabine is improved. This approach decreases the number of tumor-promoting macrophages and bolsters anti-tumor T cell responses, thereby reducing tumor growth and metastasis. Such strategies underscore the potential of integrating TAM-targeting therapies with traditional chemotherapy to better manage cancer progression (Poh and Ernst, 2021).

**FIGURE 2:**
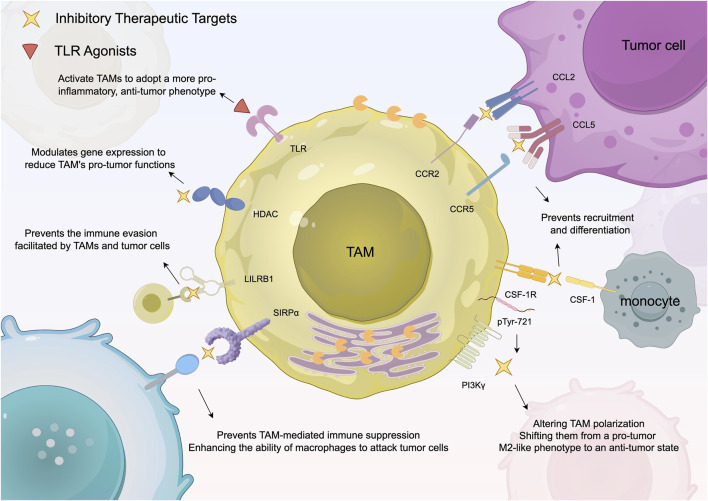
Summarizes the existing strategies for targeting TAMs to boost anti-tumor immune responses. This diagram outlines therapeutic strategies targeting TAMs in cancer treatment. It focuses on specific pathways, such as CSF-1R inhibition and CAR-M therapy, that either suppress the tumor-supporting function of M2 macrophages or reprogram them to enhance the immune response against tumors.

Oncolytic viruses are tumor-killing viruses with the ability to replicate. They invade tumor cells through cell surface molecules. One effective strategy of oncolytic virus therapy involves engineering oncolytic viruses to target specific receptors that are overexpressed in tumor cells. This allows the viruses to invade tumor cells and carry out subsequent functions, enhancing the specificity and efficacy of the therapy (Kubli et al., 2021). A recent study explored the therapeutic use of oncolytic viruses that encode IL-12, a pro-inflammatory cytokine that has previously been shown to be important for the anti-tumor ability of myeloid cells (Nguyen et al., 2020). The findings related to this are illustrated in Figure 2. After the virus particles are transported to glioblastoma, TAMs undergo a transformation to the M2 phenotype, thus killing cancer cells (Malfitano et al., 2020). The transition of TAMs to an M2 phenotype renders gliomas more susceptible to immune checkpoint inhibitors, such as anti-PD-1 and anti-CTLA-4 antibodies. Additionally, when these immune checkpoint inhibitors are administered in combination with viral therapy, they can substantially improve patient survival rates. This integrated approach presents a promising avenue for treating resistant tumors like glioma by activating and bolstering the patient’s immune response (Malfitano et al., 2020).

## 3 Conclusion and perspectives

TAMs play a pivotal yet multifaceted role in the tumor microenvironment, exhibiting both pro- and anti-tumor growth depending on their M1 or M2 polarization states (Ji et al., 2024). This complex behavior has made TAMs as significant targets for anticancer therapies aimed at reducing tumor growth, immune evasion, and metastasis. However, broad-spectrum macrophage targeting presents considerable challenges, including potential systemic toxicity that might affect healthy tissues, limited efficacy in completely controlling tumor progression, and the risk of treatment resistance. These issues necessitate the ongoing development of novel strategies to maintain therapeutic efficacy. The field is evolving with a focus on more refined targeting techniques, such as identifying TAM-specific markers or pathways for more precise intervention. Combining TAM-targeted therapies with other anticancer treatments shows promise in enhancing overall treatment effectiveness and mitigating drug resistance. An emerging strategy involves reprogramming TAMs from a tumor-promoting M2 phenotype to a tumor-inhibiting M1 phenotype, potentially reshaping the tumor microenvironment and bolstering immune responses against cancer.

As research into macrophage functions and the tumor microenvironment advances, more precise and personalized therapeutic strategies are emerging. These advancements may involve the development of highly specific treatments tailored to individual patient profiles, which aim to minimize side effects and improve outcomes. Current progress in the field is expected to address the complexities of TAM-targeted therapies more comprehensively, paving the way for more effective and individualized cancer treatments that leverage the unique dynamics of the tumor microenvironment.

In contrast to other reviews, we emphasize the integration of cutting-edge treatments, such as CAR-T cell therapies, specifically designed to target TAMs, along with the latest advancements in reprogramming macrophages from a tumor-promoting (M2-like) to a tumor-suppressing (M1-like) phenotype. Additionally, our review introduces novel mechanistic insights into the pathways regulating macrophage polarization, utilizing recent advances in single-cell RNA sequencing to provide a more granular understanding of TAM heterogeneity. The combination of these innovations, alongside a thorough examination of ongoing clinical trials and future directions for personalized therapies, ensures that this review not only summarizes current knowledge but also offers forward-looking perspectives that are underexplored in the existing literature.
